# Establishment and Evaluation of a Noninvasive Metabolism-Related Fatty Liver Screening and Dynamic Monitoring Model: Cross-Sectional Study

**DOI:** 10.2196/56035

**Published:** 2024-08-22

**Authors:** Jiali Ni, Yong Huang, Qiangqiang Xiang, Qi Zheng, Xiang Xu, Zhiwen Qin, Guoping Sheng, Lanjuan Li

**Affiliations:** 1 The First Affiliated Hospital Zhejiang University School of Medicine Hangzhou China; 2 Shulan (Hangzhou) Hospital Affiliated to Zhejiang Shuren University Shulan International Medical College Hangzhou China; 3 State Key Laboratory for Diagnosis and Treatment of Infectious Disease The First Affiliated Hospital Zhejiang University School of Medicine Hangzhou China

**Keywords:** metabolic-associated fatty liver disease, nonalcoholic fatty liver disease, nonalcoholic steatohepatitis, body fat mass, waist-height ratio, basal metabolic rate, liver

## Abstract

**Background:**

Metabolically associated fatty liver disease (MAFLD) insidiously affects people's health, and many models have been proposed for the evaluation of liver fibrosis. However, there is still a lack of noninvasive and sensitive models to screen MAFLD in high-risk populations.

**Objective:**

The purpose of this study was to explore a new method for early screening of the public and establish a home-based tool for regular self-assessment and monitoring of MAFLD.

**Methods:**

In this cross-sectional study, there were 1758 eligible participants in the training set and 200 eligible participants in the testing set. Routine blood, blood biochemistry, and FibroScan tests were performed, and body composition was analyzed using a body composition instrument. Additionally, we recorded multiple factors including disease-related risk factors, the Forns index score, the hepatic steatosis index (HSI), the triglyceride glucose index, total body water (TBW), body fat mass (BFM), visceral fat area, waist-height ratio (WHtR), and basal metabolic rate. Binary logistic regression analysis was performed to explore the potential anthropometric indicators that have a predictive ability to screen for MAFLD. A new model, named the MAFLD Screening Index (MFSI), was established using binary logistic regression analysis, and BFM, WHtR, and TBW were included. A simple rating table, named the MAFLD Rating Table (MRT), was also established using these indicators.

**Results:**

The performance of the HSI (area under the curve [AUC]=0.873, specificity=76.8%, sensitivity=81.4%), WHtR (AUC=0.866, specificity=79.8%, sensitivity=80.8%), and BFM (AUC=0.842, specificity=76.9%, sensitivity=76.2%) in discriminating between the MAFLD group and non-fatty liver group was evaluated (*P*<.001). The AUC of the combined model including WHtR, HSI, and BFM values was 0.900 (specificity=81.8%, sensitivity=85.6%; *P*<.001). The MFSI was established based on better performance at screening MAFLD patients in the training set (AUC=0.896, specificity=83.8%, sensitivity=82.1%) and was confirmed in the testing set (AUC=0.917, specificity=89.8%, sensitivity=84.4%; *P*<.001).

**Conclusions:**

The novel MFSI model was built using WHtR, BFM, and TBW to screen for early MAFLD. These body parameters can be easily obtained using a body fat scale at home, and the mobile device software can record specific values and perform calculations. MFSI had better performance than other models for early MAFLD screening. The new model showed strong power and stability and shows promise in the area of MAFLD detection and self-assessment. The MRT was a practical tool to assess disease alterations in real time.

## Introduction

Nonalcoholic fatty liver disease (NAFLD) is regarded as an important cause of liver disease, affecting more than 25% of the general population worldwide; more than 50% of patients with NAFLD also have dysmetabolism [1,2]. In 2020, experts redefined NAFLD as metabolically associated fatty liver disease (MAFLD), and much emphasis was placed on the presence of metabolic-related diseases or dysfunction [3-5]. Researchers have found that MAFLD is a multisystem disease, and liver steatosis is associated with type 2 diabetes, chronic kidney disease, cardiovascular disease, and other diseases that interact and form a vicious cycle [6-14]. China has the highest incidence of NAFLD or MAFLD morbidity in Asia [3,15,16]. Therefore, much attention should be given to MAFLD by enhancing awareness of MAFLD and optimizing its management.

To date, guidelines have suggested that liver biopsy could serve as the gold standard to diagnose histological liver damage, but noninvasive, quantitative assessment of liver fibrosis may also have prognostic implications. Ratziu et al [17] collected liver biopsy samples from 51 patients and found that 41% of the patients were at different stages of liver fibrosis or had nonalcoholic steatohepatitis. The uneven distribution of histological lesions inevitably led to sampling error when performing biopsy. Abdominal imaging, such as B-ultrasound imaging and the controlled attenuation parameter (CAP) technique, can be used to diagnosis liver disease; the former is less sensitive to mild steatosis, while the latter can detect steatosis of more than 5% and is one of the most common noninvasive methods for quantifying hepatic steatosis and fibrosis clinically [18,19]. The European Association for the Study of the Liver, European Association for the Study of Diabetes, and European Association for the Study of Obesity updated the clinical practice guidelines that propose that the nonalcoholic fatty liver disease fibrosis score (NFS) and fibrosis-4 (FIB-4) index can be used as prognostic markers for the progression of liver disease [20]. The NFS has higher specificity in the older adult population (individuals aged >65 years old) [21,22]. The predictive performance of the NFS, FIB-4 index, and aspartate aminotransferase-to-platelet ratio index (APRI) has been consistent in relation to rates of liver-related disease and mortality but is less valuable for the prediction of liver fibrosis [23]. One study found that the combination of the NFS, FIB-4 index, and liver stiffness measurement greatly improved the diagnostic accuracy, and the performance was similar to that of liver biopsy [24]. A cross-sectional study found that the triglyceride glucose (TyG) index was positively correlated with the likelihood and severity of NAFLD. The TyG index is generally considered a biomarker of steatosis, while its causal role in the judgement of fibrosis progression remains unclear [25,26]. In addition, the hepatic steatosis index (HSI) is more accurate in discriminating between MAFLD and nonfatty liver disease (non-FLD) than ultrasound. The predictive ability of the CAP for steatosis is superior to that of the HSI, and the HSI is more effective at discriminating patients with moderate-to-severe disease [18,27].

Studies have shown that numerous anthropometric indicators, such as BMI, waist-height ratio (WHtR), waist-hip ratio, and body adiposity index, are applicable for the quantification of visceral steatosis [28-32]. Body fat scales, a new popular domestic tool for health analysis, can be used to analyze basic parameters of body conditions such as the basal metabolic rate (BMR), body water distribution, and fat distribution. Reputable experts in the field have conducted extensive long-term studies on NAFLD and MAFLD, yet few noninvasive scoring models that accurately reflect disease activity or progression have been identified [33,34].

Therefore, there is an urgent need to identify more accurate predictive indicators and develop new screening methods for early MAFLD screening. The aim of this study was to construct a noninvasive prediction system for MAFLD, explore this new system for early screening in public, and establish a home-based tool for regular self-assessment and monitoring of MAFLD.

## Methods

### Study Population

**Figure 1:**
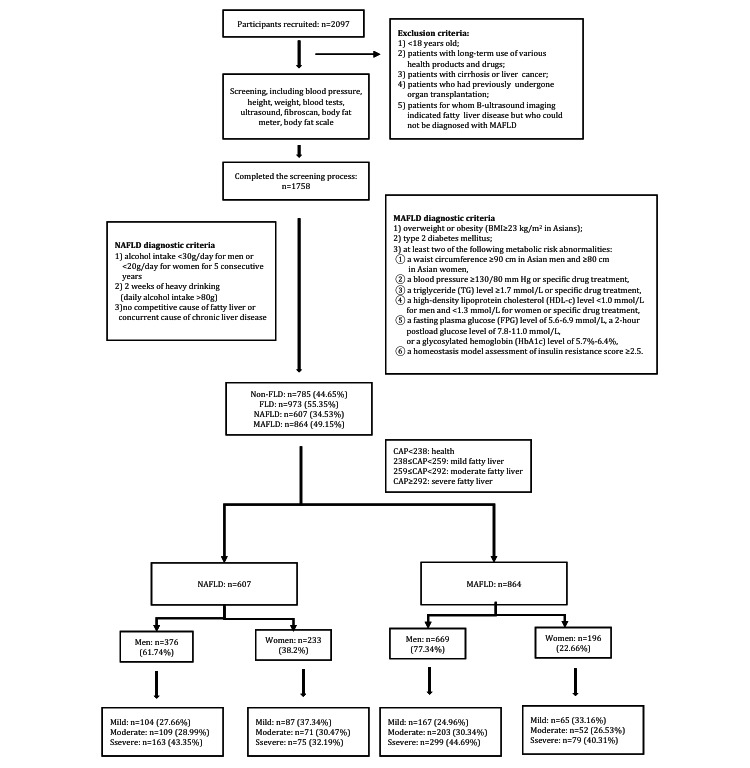
Flowchart of the inclusion process for the participants in the training set and the proportion of mild, moderate, and severe fatty liver disease in the nonalcoholic fatty liver disease (NAFLD) group and metabolically associated fatty liver disease (MAFLD) group. CAP: controlled attenuation parameter; FLD: fatty liver disease.

The participants came from Hangzhou, Shaoxing, and Quzhou from March 2021 to November 2021, and a total of 2097 participants were enrolled (Figure 1). All participants signed the informed consent form and completed the examination as required. There were 1758 eligible participants in the training set who truthfully and completely answered the questionnaire, which contained items regarding height, weight, drinking history, past medical history, and other basic information.

To validate the results of the training set, there were 200 eligible participants grouped into the testing set.

All participants were diagnosed using the liver stiffness measurement and classified according to the CAP. CA*P* values <238 was considered to indicate a healthy liver, ≥238 and <259 was considered to indicate mildly fatty liver, ≥259 and <292 was considered to indicate moderately fatty liver, and ≥292 was considered to indicate severely fatty liver [35,36].

### Exclusion Criteria

Patients who met one or more of the following criteria could not participate in this study: (1) <18 years old; (2) long-term use of various health products and drugs; (3) presence of cirrhosis or liver cancer; (4) previous organ transplantation; and (5) patients for whom B-ultrasound imaging indicated FLD but who could not be diagnosed with MAFLD.

### Diagnostic Criteria

The researchers in this study entered and organized the data, and the following diagnostic criteria were used to distinguish MAFLD patients [3]: (1) overweight or obese (BMI ≥23 kg/m^2^ in Asians), (2) presence of type 2 diabetes mellitus, and (3) at least 2 of the following metabolic risk abnormalities: waist circumference ≥90 cm in Asian men and ≥80 cm in Asian women; blood pressure ≥130/80 mm Hg or specific drug treatment; triglyceride (TG) level ≥1.7 mmol/L or specific drug treatment; high-density lipoprotein cholesterol (HDL-c) level <1.0 mmol/L for men and <1.3 mmol/L for women or specific drug treatment; fasting plasma glucose (FPG) level of 5.6 mmol/L to 6.9 mmol/L, 2-hour postload glucose level of 7.8 mmol/L to 11.0 mmol/L, or glycosylated hemoglobin level of 5.7% to 6.4%; and homeostasis model assessment of insulin resistance score ≥2.5.

### Data Collection and Model Selection

All items were completed under the guidance of the researchers. The participants underwent fasting blood tests. Waist circumference and hip circumference were measured with the participants wearing thin clothes. Body composition analysis was performed with bare feet. The patients were in a supine position during the FibroScan exam, and the right upper limb was held high and flat close to the ear. The probe was moved a small distance from the anchor point so that the most suitable detection point could be determined.

We collected basic information, including sex, age, height, weight, BMI, waist circumference, hip circumference, blood pressure, heart rate, and alcohol consumption history. The following laboratory results were included: alanine aminotransferase (ALT), aspartate aminotransferase (AST), glutamyl transpeptidase (GGT), alkaline phosphatase, hemoglobin, total cholesterol (TC), TG, HDL-c, low-density lipoprotein cholesterol (LDL-c), uric acid, and FPG levels, as well as white blood cell, red blood cell, and platelet (PLT) counts.

A body composition analyzer (InBody770, Biospace) was used to measure body composition and determine total body water (TBW), intracellular water, skeletal muscle mass, protein, and body fat mass (BFM). A body fat scale (3 Pro, Huawei) was used to determine the BMR, fat%, and visceral fat area (VFA).

The models or formulas involved in this study, including BMI, FIB-4 index, Forns index score, APRI, glutamyl transpeptidase-to-platelet ratio index (GPR), HSI, and TyG index, were developed using the following standard equations:

BMI=weight/height^2^

FIB-4=age×AST/(PLT×√ALT)

Forns index score=7.811-3.131×In PLT(109/L)+0.781×In GGT+3.467×In age-0.014×TC

APRI=(AST/upper limit of normal)/PLT×100

glutamyl transpeptidase-to-platelet ratio index=(GGT/upper limit of normal)/PLT×100

HSI=8×(ALT/AST)+BMI (female+2, diabetes+2)

TyG=ln (TG×FPG/2)

### Statistical Analysis Methods

Participants were divided into the non-FLD group, which was the healthy group; MAFLD group; and NAFLD group.

All data obtained in this study were analyzed using SPSS version 26.0 (IBM Corp). The continuous variables were tested for normality and homogeneity of variance. A *t* test was performed for measurement data that followed a normal distribution, and the results are expressed as the mean (SD). Nonnormally distributed data were analyzed using nonparametric tests, and the results are represented by quartiles. The chi-square test or Fisher precision probability test was used for quantitative data such as sex. ANOVA was followed by post hoc analysis tests to compare numerical data among the 3 groups (MAFLD, NAFLD, and non-FLD). A *P*<.01 indicated that the difference was statistically significant.

Binary logistic regression analysis was performed to explore the potential anthropometric indicators with predictive ability to screen for MAFLD. A receiver operating characteristic (ROC) curve was drawn based on the selected indicators, and the area under the ROC curve (AUC) was calculated correspondingly. The indicator with the highest AUC was considered the most valuable indicator. The maximum Youden index (using the formula sensitivity + specificity - 1) was used to define the optimal cutoff value. Potential confounding variables were added into the logistic regression equation step by step, including age; blood pressure; and FPG, TC, TG, HDL-c, and LDL-c levels. Calibration Model I (age, blood pressure, and FPG level were added to the logistic regression equation) and Model II (age; blood pressure; and FPG, TC, TG, HDL-c, and LDL-c levels were added to the logistic regression equation) were established, and the predictive ability was evaluated before and after calibration. All significant indicators were included for the combination of diagnostic tests, and ROC curves were drawn. A new prediction model, the MAFLD Screening Index (MFSI), was constructed using logistic regression analysis, and the model was validated with the testing set. All tests were 2-tailed, and *P*<.01 was considered statistically significant.

### Ethical Considerations

Every participant signed a written informed consent form and participated in the study anonymously. We ensured it was not possible to identify individual participants in any images used in manuscripts or other materials.

Every participant was given an allowance of ¥300 (US $0.14) upon completion of the research project. The study protocol was approved by the Ethics Committee of Shulan Hangzhou Hospital (approval number KY2021001).

The study did not involve additional invasive procedures, and there were no associated adverse reactions.

## Results

### Comparing Numerical Data Among the 3 Groups (MAFLD, NAFLD, and Non-FLD)

Using ANOVA to compare the basic information and anthropometric indicators among the 3 groups, the results showed that all parameters were significantly different among the MAFLD, NAFLD, and non-FLD groups. After the post hoc analysis, PLT count (*P*=.10) was not significantly different between the MAFLD and non-FLD groups, and white blood cell count (*P*=.26), TC (*P*=.35), LDL-c (*P*=.11), VFA (*P*=.07), and Fat% (*P*=.38) were not significantly different between the MAFLD and NAFLD groups (Table 1).

**Table 1:** Baseline characteristics and anthropometric indicators compared among 3 groups: metabolically associated fatty liver disease (MAFLD), nonalcoholic fatty liver disease (NAFLD), and non-fatty liver disease (non-FLD)

Characteristics			non-FLD (n=786)		MAFLD (n=864)		NAFLD (n=607)		Statistic (*df*)			*P* value	
**Sex, n (%)**										224.985^a^ (2)			<.001
	Male	326 (41.5)		668 (77.3)		375 (61.8)							
	Female	460 (58.5)		196 (22.7)		232 (38.2)							
Age (years), mean (range)			36 (28-48)		45 (34-55)		40 (31-53)		107.212^b^ (2)			<.001	
Height (cm), mean (SD)			164.17 (7.820)		168.23 (7.844)		166.81 (8.358)		54.873^c^			<.001	
Weight (kg), mean (range)			57.30 (51.60-64.30)		72.9 (66.2-80.7)		69.7 (61.1-78.1)		664.463^b^ (2)			<.001	
BMI (kg/m^2^), mean (range)			21.49 (20.06-23.18)		25.66 (24.04-27.79)		25.04 (22.99-2t7.12)		798.113^b^ (2)			<.001	
SBP^d^ (mm Hg), mean (range)			119 (110-135)		132 (121-144)		128 (118-140)		218.758^b^ (2)			<.001	
DBP^e^ (mm Hg), mean (SD)			74.89 (11.216)		82.88 (11.863)		79.72 (11.611)		91.652^c^ (2,2249)			<.001	
WBC^f^ (109/L), mean (range)			5.80 (4.95-6.80)		6.5 (5.5-7.6)		6.3 (5.4-7.5)		91.944^b^ (2)			<.001	
RBC^g^ (1012/L), mean (range)			4.69 (4.39-5.11)		5.12 (4.81-5.42)		5.02 (4.65-5.38)		210.018^b^ (2)			<.001	
Hb^h^ (g/L), mean (range)			140 (131-153)		155 (145-163)		150 (137-160)		213.919^b^ (2)			<.001	
PLT^i^ (109/L), mean (SD)			234.66 (55.331)		238.18 (58.518)		244.67 (58.671)		5.041^c^ (2,2091)			.006	
FPG^j^ (mmol/L), mean (range)			4.60 (4.36-4.89)		4.88 (4.55-5.34)		4.80 (4.50-5.19)		128.314^b^ (2)			<.001	
ALT^k^ (U/L), mean (range)			14 (10-20)		27.00 (19.00-34.00)		24.00 (17.75-40.00)		475.850^b^ (2)			<.001	
AST^l^ (U/L), mean (range)			20 (17-24)		25.00 (21.00-32.00)		24.00 (19.00-29.00)		245.274^b^ (2)			<.001	
ALP^m^ (U/L), mean (range)			57 (47-70)		69.00 (57.00-83.00)		66.00 (55.00-81.00)		146.960^b^ (2)			<.001	
GGT^n^ (U/L), mean (range)			15 (12-21)		31.00 (20.00-52.00)		24.00 (17.00-38.00)		496.679^b^ (2)			<.001	
TC^o^ (mmol/L), mean (range)			4.71 (4.13-5.23)		4.99 (4.38-5.33)		4.96 (4.33-5.55)		47.740^b^ (2)			<.001	
TG^p^ (mmol/L), mean (range)			0.93 (0.71-1.26)		1.76 (1.21-2.45)		1.49 (1.07-2.24)		529.457^b^ (2)			<.001	
HDL-c^q^ (mmol/L), mean (range)			1.48 (1.28-1.68)		1.21 (1.07-1.40)		1.26 (1.07-1.45)		284.363^b^ (2)			<.001	
LDL-c^r^ (mmol/L), mean (range)			2.64 (2.23-3.16)		3.10 (2.64-3.64)		3.04 (2.56-3.55)		146.926^b^ (2)			<.001	
UA^s^ (μmol/L), mean (SD)			295.99 (80.86)		378.82 (85.081)		358.39 (87.676)		206.430^c^ (2,2234)			<.001	
E-value (kPa), mean (range)			4.40 (3.70-5.20)		5.2 (4.3-6.3)		5.0 (4.2-6.0)		164.275^b^ (2)			<.001	
CAP^t^ (dB/m), mean (range)			200 (179-218)		284 (257-320)		278 (255-315)		1538.285^b^ (2)			<.001	
WHtR^u^, mean (SD)			0.459 (0.0656)		0.524 (0.5117)		0.512 (0.0984)		113.769^c^			<.001	
WHR^v^, mean (range)			0.829 (0.783-0.880)		0.918 (0.872-0.950)		0.892 (0.840-0.939)		284.420^b^ (2)			<.001	
FIB-4^w^ index, mean (range)			0.813 (0.588-1.234)		0.954 (0.608-1.362)		0.782 (0.521-1.235)		15.698^b^ (2)			<.001	
Forns index, mean (SD)			5.471 (1.6797)		6.536 (1.5977)		6.005 (1.6630)		166.437^c^ (2,2190)			<.001	
APRI^x^, mean (range)			0.240 (0.187-0.301)		0.282 (0.215-0.376)		0.263 (0.201-0.351)		72.070^b^ (2)			<.001	
GPR^y^, mean (range)			0.133 (0.103-0.192)		0.230 (0.159-0.403)		0.186 (0.135-0.292)		325.345^b^ (2)			<.001	
HSI^z^, mean (rang)			28.57 (26.31-30.84)		35.28 (32.02-39.04)		34.47 (30.87-38.63)		772.797^b^ (2)			<.001	
TyG^aa^ index, mean (range)			8.137 (7.863-8.463)		8.855 (8.463-9.234)		8.674 (8.322-9.092)		585.116^b^ (2)			<.001	
TBW^bb^ (kg), mean (range)			30.30 (27.2-036.40)		38.65 (33.80-42.90)		36.85 (30.23-42.88)		341.198^b^ (2)			<.001	
ICW^cc^, mean (range)			18.80 (16.70-22.60)		24.10 (20.90-26.80)		22.80 (18.70-26.10)		340.014^b^ (2)			<.001	
Protein, mean (range)			8.10 (7.20-9.80)		10.40 (9.08-11.60)		9.90 (8.10-11.30)		339.536^b^ (2)			<.001	
BFM^dd^, mean (range)			14.90 (12.00-17.40)		20.80 (17.60-24.43)		20.10 (16.80-24.20)		638.768^b^ (2)			<.001	
SMM^ee^, mean (range)			22.50 (19.80-27.53)		29.40 (25.30-32.93)		27.80 (22.35-32.10)		336.593^b^ (2)			<.001	
BMR^ff^ (kcal), mean (range)			1283.95 (1179.70-1450.17)		1526.55 (1387.09-1655.52)		1470.15 (1276.65-1622.62)		358.763^b^ (2)			<.001	
VFA^gg^, mean (range)			6.552 (5.235-7.762)		9.013 (7.598-10.813)		8.753 (7.294-10.671)		518.559^b^ (2)			<.001	
Fat (%), mean (SD)			25.319 (5.9566)		28.255 (5.2256)		28.524 (5.7358)		68.099^c^ (2,2018)			<.001	

^a^Chi-squared test.

^b^*H* value.

^c^*F* value.

^d^SBP: systolic blood pressure.

^e^DBP: diastolic blood pressure.

^f^WBC: white blood cell.

^g^RBC: red blood cell.

^h^Hb: hemoglobin.

^i^PLT: platelet.

^j^FPG: fasting plasma glucose.

^k^ALT: alanine aminotransferase.

^l^AST: aspartate aminotransferase.

^m^ALP: alkaline phosphatase.

^n^GGT: glutamyl transpeptidase.

^o^TC: total cholesterol.

^p^TG: triglyceride.

^q^HDL-c: high-density lipoprotein cholesterol.

^r^LDL-c: low-density lipoprotein cholesterol.

^s^UA: uric acid.

^t^CAP: controlled attenuation parameter.

^u^WHtR: waist-height ratio.

^v^WHR: waist-hip ratio.

^w^FIB-4: fibrosis-4.

^x^APRI: aspartate aminotransferase-to-platelet ratio index.

^y^GPR: glutamyl transpeptidase-to-platelet ratio index.

^z^HSI: hepatic steatosis index.

^aa^TyG: triglyceride glucose.

^bb^TBW: total body water.

^cc^ICW: intracellular water.

^dd^BFM: body fat mass.

^ee^SMM: skeletal muscle mass.

^ff^BMR: basal metabolic rate.

^gg^VFA: visceral fat area.

### Predictive Performance of Different Anthropometric Indicators

**Figure 2:**
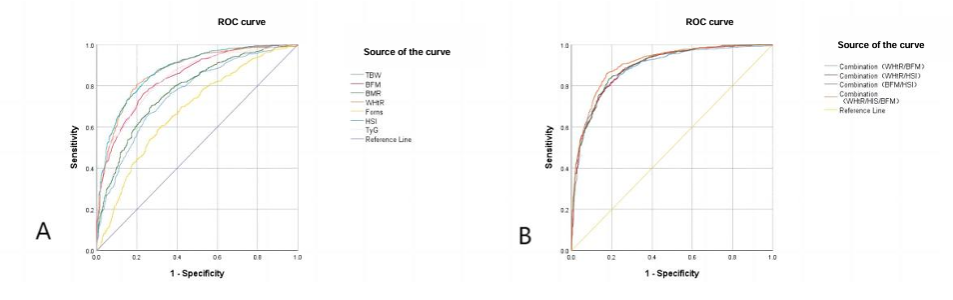
Receiver operating characteristic (ROC) curves for the screening ability of different anthropometric indicators for metabolically associated fatty liver disease (MAFLD): (A) screening ability of the waist-height ratio (WHtR), Frons index, hepatic steatosis index (HSI), triglyceride glucose (TyG) index, total body water (TBW), body fat mass (BFM), basal metabolic rate (BMR) and (B) screening ability of combinations of WHtR, HSI, and BFM. Diagonal segments were produced by ties.

The variables in the previous section with a *P*<.01 were further included in the logistic regression analysis. The ROC curves and optimal cutoff points for the selected indicators are shown in Table 2 and Figure 2. The AUCs of the WHtR, the Forns index, the HSI, the TyG index, TBW, BFM, and BMR were 0.866, 0.684, 0.873, 0.835, 0.760, 0.842, and 0.778, respectively (*P*<.001; Table 2).

**Table 2:** Cutoff points and areas under the curve (AUCs) were used to demonstrate the screening ability of the different anthropometric indicators for metabolically associated fatty liver disease (n=1649).

Anthropometric indicators	AUC (95% CI)	*P* value	Cutoff point	Specificity (%)	Sensitivity (%)
WHtR^a^	0.866	<.001	0.501449713	79.8	80.8
Forns index	0.684	<.001	6.160599276	67.8	61.3
HSI^b^	0.873	<.001	31.15061285	76.8	81.4
TyG^c^ index	0.835	<.001	8.450341708	74	76.5
TBW^d^ (kg)	0.760	<.001	36.55	75.3	65.3
BFM^e^	0.842	<.001	17.55	76.9	76.2
BMR^f^	0.778	<.001	1434.263124	73.1	70.1
Combination (WHtR/HSI)	0.885	<.001	N/A^g^	76	85.6
Combination (WHtR/BFM)	0.881	<.001	N/A	81.7	80
Combination (BFM/HSI)	0.889	<.001	N/A	81	82.9
Combination (WHtR/HSI/BFM)	0.900	<.001	N/A	81.8	85.6

^a^WHtR: waist-to-hip ratio.

^b^HSI: hepatic steatosis index.

^c^TyG: triglyceride glucose.

^d^TBW: total body water.

^e^BFM: body fat mass.

^f^BMR: basal metabolic rate.

^g^N/A: not applicable.

According to the ROC curve and AUC (Figure 2), HSI had the strongest predictive performance for MAFLD in the training set, and the performance ranking was as follows: HSI, WHtR, BFM, TyG index, TBW, and Forns index. The confounding factors were further corrected for in the logistic regression analysis (Model I: age, blood pressure, and FPG level were added to the logistic regression equation; Model II: age; blood pressure; and FPG, TC, TG, HDL-c, and LDL-c levels were added to the logistic regression equation). After correction for confounding factors, the odds ratio (OR) of the Forns index in Model I was 1.043 (95% CI 0.851-1.277), and that in Model II was 1.050 (95% CI 0.854-1.293; Table 3). The results showed that the performance of the Forns index for MAFLD screening was unstable and the performance of other anthropometric indicators was not easily influenced by confounders.

The HSI and WHtR showed better predictive performance than the other indicators. The sensitivity of the HSI was higher than that of the other anthropometric indicators (sensitivity=81.4%), and the specificity of the WHtR was higher than that of the other anthropometric indicators (specificity=79.8%). The combination of WHtR, HSI, and BFM increased the predictive ability for MAFLD, and the AUC was 0.900 (specificity=81.8%, sensitivity=85.6%; *P*<.001; Table 2).

**Table 3:** Confounders were corrected in the binary logistic regression analysis to compare changes in screening power for different anthropometric indicators (n=1649).

Anthropometric indicators	Nonadjusted model		Model I			Model II	
	OR^a^ (95% CI)	*P* value	OR (95% CI)	*P* value	OR (95% CI)		*P* value
TBW^b^ (kg)	1.079 (1.051-1.109)	<.001	1.086 (1.055-1.118)	<.001	1.075 (1.042-1.108)		<.001
BFM^c^	1.255 (1.194-1.319)	<.001	1.250 (1.186-1.317)	<.001	1.257 (1.192-1.326)		<.001
WHtR^d^	145.540 (9.015-2349.524)	<.001	107.825 (6.232-1865.456)	.001	113.408 (6.759-1902.900)		<.001
Forns index	1.166 (1.053-1.292)	.003	1.043 (0.851-1.277)	.69	1.050 (0.854-1.293)		.64
HSI^e^	1.124 (1.070-1.182)	<.001	1.128 (1.070-1.190)	<.001	1.110 (1.052-1.172)		<.001
TyG^f^ index	6.557 (4.560-9.427)	<.001	5.832 (3.972-8.562)	<.001	6.005 (3.764-9.579)		<.001

^a^OR: odds ratio.

^b^TBW: total body water.

^c^BFM: body fat mass.

^d^WHtR: waist-height ratio.

^e^HSI: hepatic steatosis index.

^f^TyG: triglyceride glucose.

### Development of a New MAFLD Screening Model

**Figure 3:**
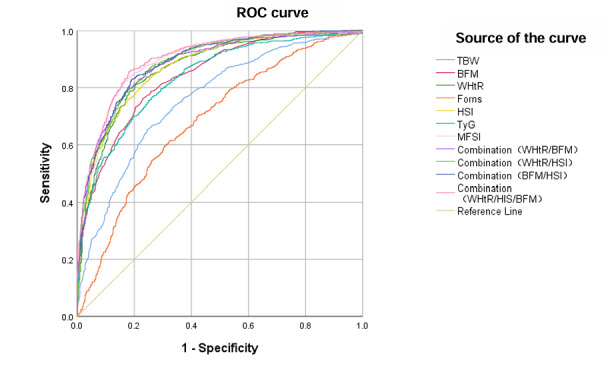
Receiver operator characteristic (ROC) curves showing the screening ability of different combinations of anthropometric indicators and a new metabolically associated fatty liver disease (MAFLD) screening model named the MAFLD screening index (MFSI=–13.968+0.120×total body water [TBW]+0.254×body fat mass [BFM]+10.793×waist-height ratio [WHtR]). Diagonal segments were produced by ties. BMR: basal metabolic rate; HSI: hepatic steatosis index; TyG: triglyceride glucose.

The HSI, WHtR, and BFM displayed strong power in screening for MAFLD. The HSI was calculated based on BMI, ALT levels, and AST levels and was not suitable for early screening for MAFLD. The purpose of establishing a new model was to reduce the need for invasive procedures and reduce the frequency of medical visits, as well as to screen for MAFLD in high-risk populations. The predictive ability of TBW was stable after correcting for confounders (Model I: 95% CI 1.055-1.118; Model II: 95% CI 1.042-1.108; Table 3). Therefore, TBW was included in the new model. Logistic regression analysis was used to establish the MAFLD early screening model, which was named the MFSI. The formula was as follows: MFSI=–13.968+0.120×TBW+0.254×BFM+10.793×WHtR (Figure 3). The AUC of the MFSI was 0.896 (specificity: 83.8%, sensitivity: 82.1%; *P*<.001; Table 4). Collectively, the performance of the MFSI and the WHtR/HSI/BFM combination models was similar.

**Table 4:** Cutoff points and areas under the curve (AUCs) were used to compare the screening ability of different anthropometric indicators and the metabolically associated fatty liver disease screening index (MFSI; n=1649).

Anthropometric indicators	AUC (95% CI)	*P* value	Cutoff point	Specificity (%)	Sensitivity (%)
WHtR^a^	0.866	<.001	0.501449713	79.8	80.8
Forns index	0.684	<.001	6.160599276	67.8	61.3
HSI^b^	0.873	<.001	31.15061285	76.8	81.4
TyG^c^ index	0.835	<.001	8.450341708	74	76.5
TBW^d^ (kg)	0.760	<.001	36.55	75.3	65.3
BFM^e^	0.842	<.001	17.55	76.9	76.2
Combination (WHtR/HSI)	0.885	<.001	N/A^f^	76	85.6
Combination (WHtR/BFM)	0.881	<.001	N/A	81.7	80
Combination (BFM/HSI)	0.889	<.001	N/A	81	82.9
Combination (WHtR/HSI/BFM)	0.900	<.001	N/A	81.8	85.6
MFSI	0.896	<.001	0.5146795	83.8	82.1

^a^WHtR: waist-height ratio.

^b^HSI: hepatic steatosis index.

^c^TyG: triglyceride glucose.

^d^TBW: total body water.

^e^BFM: body fat mass.

^f^N/A: not applicable.

### Performance of the MFSI in the Testing Set

**Figure 4:**
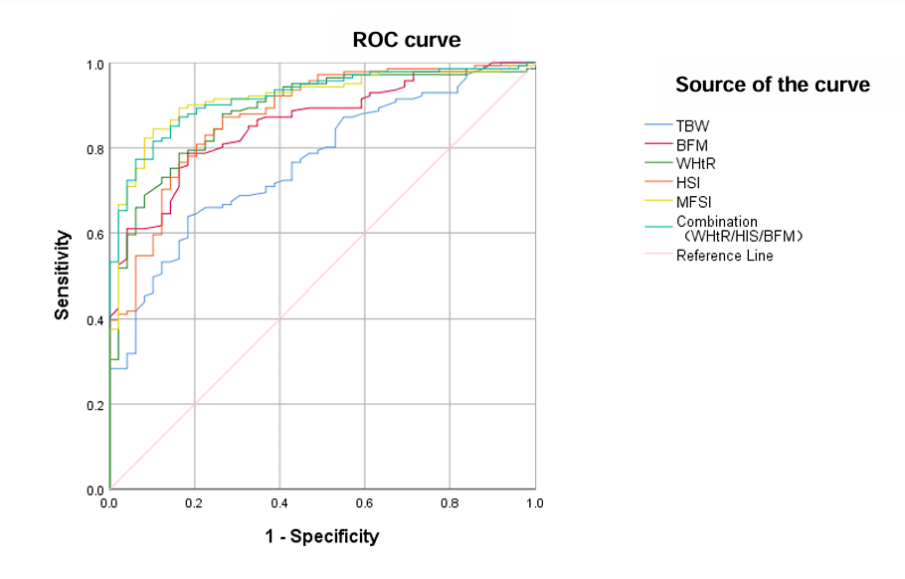
Receiver operating characteristic (ROC) curves for the screening ability of different anthropometric indicators and metabolically associated fatty liver disease screening index (MFSI) in the testing set. Diagonal segments were produced by ties. BFM: body fat mass; HSI: hepatic steatosis index; TBW: total body water; WHtR: waist-height ratio.

There were a further 200 participants enrolled in the testing set, including 51 non-FLD patients and 149 MAFLD patients. To evaluate the predictive ability of the MFSI for screening for MAFLD in a high-risk population, the MFSI was used with the testing set, and ROC curves were drawn based on the MFSI; BFM; WHtR; HSI; TBW; and the combined model with WHtR, HSI, and BFM (Figure 4). The AUC (testing set) of the MFSI was 0.917, the specificity was 89.8%, and the sensitivity was 84.4%. The AUC (testing set) of the combined model with WHtR, HSI, and BFM was 0.920, the specificity was 89.8%, and the sensitivity was 81.6% (Table 5). The performance of the MFSI was similar to that of the combined model with WHtR, HSI, and BFM in the testing set.

**Table 5:** Areas under the curve (AUCs) were used to compare the ability of the different anthropometric indicators and the metabolically associated fatty liver disease (MAFLD) screening index (MFSI) to screen for MAFLD in the testing set (n=200).

Anthropometric indicators	AUC (95% CI)	*P* value	Specificity (%)	Sensitivity (%)
WHtR^a^	0.886	<.001	83.7	78.7
TBW^b^ (kg)	0.767	<.001	81.6	63.8
BFM^c^	0.858	<.001	81.6	78.7
HSI^d^	0.877	<.001	73.5	87.2
Combination (WHtR/HSI/BFM)	0.920	<.001	89.8	81.6
MFSI	0.917	<.001	89.8	84.4

^a^WHtR: waist-height ratio.

^b^TBW: total body water.

^c^BFM: body fat mass.

^d^HSI: hepatic steatosis index.

### MAFLD Rating Table for Prediction of MAFLD

**Figure 5:**
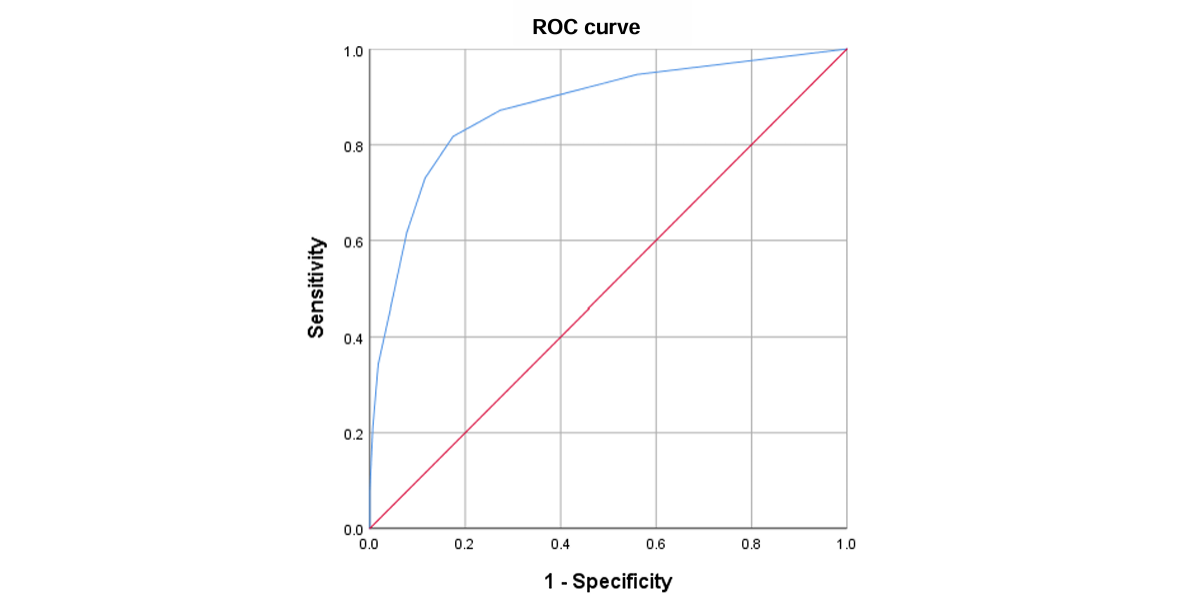
Receiver operating characteristic (ROC) curves of the correlation between the metabolically associated fatty liver disease (MAFLD) Rating Table (MRT) and MAFLD. Diagonal segments were produced by ties.

The scoring system based on the MFSI and the application program was more practical for patient self-assessment. The MAFLD Rating Table (MRT) also included TBW, BFM, and WHtR. An MRT score ranging from 0 to 2 indicated a healthy individual, and a score ≥3 indicated MAFLD (Table 6). The AUC of the MRT for MAFLD prediction was 0.876 (*P*<.001; Figure 5).

**Table 6:** Simple rating table to assess risk factors for metabolically associated fatty liver disease (MAFLD).

Factors	Rating				
	0	1	2	3	4
TBW^a^ (kg)	<33.35	33.35-45.05	≥45.05	N/A^b^	N/A
BFM^c^	<17.55	17.55-20.15	10.15-22.95	≥22.95	N/A
WHtR^d^	<0.501	N/A	0.501-0.525	0.525-0.538	≥0.538

^a^TBW: total body water.

^b^N/A: not applicable.

^c^BFM: body fat mass.

^d^WHtR: waist-to-height ratio.

## Discussion

WHtR, BFM, and TBW were predictors of MAFLD. The AUC of the WHtR was 0.866 (specificity=79.8%, sensitivity=80.8%), the AUC of BFM was 0.842 (specificity=76.9%, sensitivity=76.2%), and the AUC of TBW was 0.760 (specificity=75.3%, sensitivity=65.3%). The novel MFSI model, derived through logistic regression analysis, included the WHtR, BFM, and TBW. Notably, the MFSI demonstrated independence from laboratory findings. Upon validation, the MFSI exhibited stability while offering advantages in terms of sensitivity and specificity for MAFLD screening (training set: AUC=0.896, specificity=83.8%, sensitivity=82.1%; testing set: AUC=0.917, specificity=89.8%, sensitivity=84.4%).

Researchers found that the measurement of visceral fat can predict the occurrence of chronic diseases, such as diabetes, hyperuricemia, and metabolic syndrome [37,38]. NAFLD and MAFLD affect more than 25% of the global population and are considered different stages of the disease course. Because of long-term subtle inflammation and unobvious clinical manifestations, some patients gradually develop liver fibrosis and cirrhosis [1]. It is important to raise awareness within the population and optimize the management of this disease.

In recent decades, researchers have considered that the APRI, FIB-4 index, BMI, HSI, and TyG index have high accuracy for the diagnosis of liver fibrosis. Nonfibrosis scores were higher in patients with MAFLD than in those with NAFLD [11,39-42]. Similar conclusions were drawn in this work. To distinguish patients with MAFLD in our study population, we compared traditional indicators and body composition between the MAFLD and non-FLD groups and found there was a significant difference between the 2 groups. Although traditional indicators had efficient performance for the prediction of liver fibrosis, it was doubtful that these indicators were robust for the screening of MAFLD before being confirmed by histological liver examination. Previously published work mainly focused on the predictive ability of indicators for the detection of liver fibrosis, while much work omitted the performance of these indicators for the early screening of MAFLD.

Lee et al [27] suggested a new indicator named the HSI, and they found that NAFLD cannot be diagnosed when the HSI was <30.0, with a sensitivity of 92.5% (95% CI 91.4-93.5) The HSI showed similar performance for MAFLD diagnosis in this study. Italian researchers proposed another new indicator, the fatty liver index (FLI), which is calculated based on waist circumference, BMI, TG levels, and GGT levels. When the FLI is <30, a diagnosis of FLD can be ruled out, and when the FLI is ≥60, patients can be diagnosed with FLD. Waist circumference and BMI are the most robust predictors for the screening of FLD [43]. In contrast to the HSI, the FLI was established by incorporating waist circumference. However, BMI and waist circumference are totally different for people with varied dietary habits, and the study did not take this into account. Zheng et al [44] found that the WHtR had great performance for MAFLD screening, with a sensitivity of 96% and specificity of 64%. In our study, the WHtR showed a sensitivity of 80.8% and specificity of 79.8% for MAFLD screening.

TG and FPG levels are considered 2 pivotal inducers of metabolic syndrome. TGs are produced excessively in the process of fat accumulation, and insulin resistance accelerates hepatic steatosis. The TyG index can be used as a simple alternative marker for the detection of insulin resistance in the diagnostic test combining TG and FPG levels. The prevalence and severity of MAFLD are positively correlated with the TyG index [25,45-47]. The AUC of the TyG index for predicting MAFLD was 0.835 (95% CI 4.560-9.427), which might be valuable for clinical practice.

A meta-analysis revealed that the visceral adiposity index was an independent predictor of MAFLD, which could be used to predict potential morbidity [48]. However, the predictive ability of the visceral adiposity index has not been verified. Wang et al [49] found that nonobese MAFLD patients had higher BFM and VFA values than the healthy population, and most of them had abnormal lipid metabolism. In addition, BFM and VFA were valuable for distinguishing MAFLD patients from nonobese people [49,50]. This conclusion was also confirmed in this study (BFM for the prediction of MAFLD: AUC=0.842, sensitivity=76.2%, specificity=76.9%).

This study aimed to establish a home-based model for early screening of MAFLD to promote disease self-assessment and management. Compared with previously published models that rely heavily on laboratory indicators, our model combined body composition and the WHtR to screen for MAFLD, and the body parameters that were used to build the screening model can be easily obtained using a body fat scale at home. The mobile device software can record specific values and perform calculations.

There were 2 significant advantages of our model: (1) The need for an invasive examination and medical expenditures were reduced; (2) early screening models can provide early warning signs of disease, prompting people to modify diet and exercise or seek medical treatment if necessary; (3) patient-physician interactions were enhanced.

There were also some limitations of our work. First, this study was limited by geographical factors, and regional bias existed. Second, due to ethical considerations, the results in this study cannot be confirmed by histological liver examination. Third, in some villages we went to for recruitment, we were unable to obtain a radiological diagnosis due to manpower, transportation, and other constraints. In addition, it was difficult to follow participants who underwent physical examination in different areas, and reexamination data could not be compared with previous data.

Although our study found that the new MFSI model and MRT were valuable for MAFLD prediction, disease diagnosis still requires experienced clinicians, and those with the disease or at high risk should seek timely medical attention.

## Abbreviations

ALT: alanine aminotransferase
APRI: aspartate aminotransferase-to-platelet ratio index
AST: aspartate aminotransferase
AUC: area under the curve
BFM: body fat mass
BMR: basal metabolic rate
CAP: controlled attenuation parameter
FIB-4: fibrosis-4
FLD: fatty liver disease
FLI: fatty liver index
FPG: fasting plasma glucose
GGT: glutamyl transpeptidase
GPR: glutamyl transpeptidase-to-platelet ratio index
HDL-c: high-density lipoprotein cholesterol)
HSI: hepatic steatosis index
LDL-c: low-density lipoprotein cholesterol
MAFLD: metabolically associated fatty liver disease
MFSI: MAFLD screening index
MRT: MAFLD Rating Table
NAFLD: nonalcoholic fatty liver disease
NFS: nonalcoholic fatty liver disease fibrosis score
OR: odds ratio
PLT: platelet
ROC: receiver operating characteristic
TBW: total body water
TC: total cholesterol
TG: triglyceride
TyG: triglyceride glucose
VFA: visceral fat area
WHtR: waist-height ratio

## Data Availability

The data are not publicly available due to cooperative project clauses. Please contact the author to inquire if the data in this study are available for other studies.
